# *N*-Benzoyl-*N*-(3-methyl­phen­yl)-*O*-[2-(2-nitro­phen­yl)acet­yl]hydroxyl­amine

**DOI:** 10.1107/S1600536811025864

**Published:** 2011-07-09

**Authors:** Kai Zhang, Dian He

**Affiliations:** a Institute of Medicinal Chemistry School of Pharmacy, Lanzhou University, Lanzhou 730000, Gansu Province, People’s Republic of China

## Abstract

In the title mol­ecule, C_22_H_18_N_2_O_5_, the nitro-substituted ring makes a dihedral angle of 81.9 (1)° with the benzoyl ring and a dihedral angle of 12.1 (1)° with the methyl-substituted ring.

## Related literature

For applications, see: Zeng *et al.* (2003 ▶). For the preparation, see: Ayyangark *et al.* (1986 ▶). 
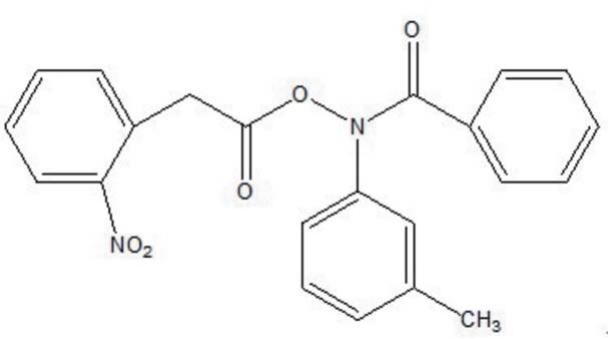


## Experimental

### 

#### Crystal data

C_22_H_18_N_2_O_5_
*M* *_r_* = 390.38
Monoclinic, 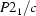

*a* = 16.34 (2) Å
*b* = 8.459 (10) Å
*c* = 14.862 (18) Å
β = 109.869 (11)°
*V* = 1932 (4) Å^3^
*Z* = 4
Mo *K*α radiation
μ = 0.10 mm^−1^
*T* = 296 K
0.25 × 0.24 × 0.21 mm

#### Data collection

Bruker APEXII CCD diffractometer
Absorption correction: multi-scan (*SADABS*; Sheldrick, 1996 ▶) *T*_min_ = 0.976, *T*_max_ = 0.980
10929 measured reflections
3591 independent reflections
2242 reflections with *I* > 2σ(*I*)
*R*_int_ = 0.047

#### Refinement

*R*[*F* ^2^ > 2σ(*F* ^2^)] = 0.050
*wR*(*F* ^2^) = 0.123
*S* = 1.00
3591 reflections
263 parameters
H-atom parameters constrained
Δρ_max_ = 0.18 e Å^−3^
Δρ_min_ = −0.21 e Å^−3^

### 

Data collection: *APEX2* (Bruker, 2009 ▶) ; cell refinement: *SAINT* (Bruker, 2009 ▶); data reduction: *SAINT*; program(s) used to solve structure: *SHELXS97* (Sheldrick, 2008 ▶); program(s) used to refine structure: *SHELXL97* (Sheldrick, 2008 ▶); molecular graphics: *SHELXTL* (Sheldrick, 2008 ▶); software used to prepare material for publication: *SHELXL97*.

## Supplementary Material

Crystal structure: contains datablock(s) global, I. DOI: 10.1107/S1600536811025864/ld2012sup1.cif

Structure factors: contains datablock(s) I. DOI: 10.1107/S1600536811025864/ld2012Isup2.hkl

Supplementary material file. DOI: 10.1107/S1600536811025864/ld2012Isup3.cml

Additional supplementary materials:  crystallographic information; 3D view; checkCIF report

## Figures and Tables

**Fig. 1.**
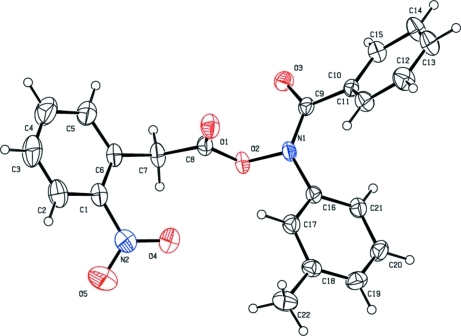
The molecular structure of the title compound with the atomic numbering and 50% probability displacement dllipsoids. H atoms are shown as small spheres of arbitrary radius.

## supplementary crystallographic information

### Comment

Hydroxamic acid derivatives have received considerable attention in recent years as the result of the discovery of their role in the biochemical toxicology of many drugs and other chemicals. Thus, these compounds continue to attract much attention as potential biological agents. The title molecule, C_22_H_18_N_2_O_5_, contains three branched chains with its centre placed at midpoint of the N. The phenyl ring C1—C6 makes a dihedral angle of 81.85 (8)° with the phenyl ring C10—C15 of benzoyl group, and 12.08 (8)° with the penyl ring C16—C21.

### Experimental

The title compound, C_22_H_18_N_2_O_5_ was prepared according to the method described by Ayyangark *et al.* (1986). Crystals of (I) suitable for single-crystal X-ray analysis were grown by slow evaporation of a solution in dichloromethane-methanol (1:3 *v*/*v*).

### Refinement

The methyl hydrogen atoms were positioned geometrically (AFIX 137) and refined using a riding/rotating model, with *U*_iso_ = 1.5 times *U*_eq_(C). Other H-atoms were positioned geometrically and allowed to ride on their parent atoms, with C—H=0.95 Å and *U*_iso_ = 1.2 times *U*_eq_(C).

### Crystal data

**Table:**

C_22_H_18_N_2_O_5_	*F*(000) = 816
*M**_r_* = 390.38	*D*_x_ = 1.342 Mg m^−^^3^
Monoclinic, *P*2_1_/*c*	Mo *K*α radiation, λ = 0.71073 Å
Hall symbol: -P 2ybc	Cell parameters from 2435 reflections
*a* = 16.34 (2) Å	θ = 2.7–22.3°
*b* = 8.459 (10) Å	µ = 0.10 mm^−^^1^
*c* = 14.862 (18) Å	*T* = 296 K
β = 109.869 (11)°	Block, yellow
*V* = 1932 (4) Å^3^	0.25 × 0.24 × 0.21 mm
*Z* = 4	

### Data collection

**Table:**

Bruker APEXII CCD diffractometer	3591 independent reflections
Radiation source: fine-focus sealed tube	2242 reflections with *I* > 2σ(*I*)
graphite	*R*_int_ = 0.047
φ and ω scans	θ_max_ = 25.5°, θ_min_ = 2.7°
Absorption correction: multi-scan (*SADABS*; Sheldrick, 1996)	*h* = −19→15
*T*_min_ = 0.976, *T*_max_ = 0.980	*k* = −10→10
10929 measured reflections	*l* = −18→18

### Refinement

**Table:**

Refinement on *F*^2^	0 restraints
Least-squares matrix: full	H-atom parameters constrained
*R*[*F*^2^ > 2σ(*F*^2^)] = 0.050	*w* = 1/[σ^2^(*F*_o_^2^) + (0.0508*P*)^2^ + 0.3323*P*] where *P* = (*F*_o_^2^ + 2*F*_c_^2^)/3
*wR*(*F*^2^) = 0.123	(Δ/σ)_max_ < 0.001
*S* = 1.00	Δρ_max_ = 0.18 e Å^−^^3^
3591 reflections	Δρ_min_ = −0.21 e Å^−^^3^
263 parameters	

### Special details

**Table:**

Geometry. All e.s.d.'s (except the e.s.d. in the dihedral angle between two l.s. planes) are estimated using the full covariance matrix. The cell e.s.d.'s are taken into account individually in the estimation of e.s.d.'s in distances, angles and torsion angles; correlations between e.s.d.'s in cell parameters are only used when they are defined by crystal symmetry. An approximate (isotropic) treatment of cell e.s.d.'s is used for estimating e.s.d.'s involving l.s. planes.
Refinement. Refinement of *F*^2^ against ALL reflections. The weighted *R*-factor *wR* and goodness of fit *S* are based on *F*^2^, conventional *R*-factors *R* are based on *F*, with *F* set to zero for negative *F*^2^. The threshold expression of *F*^2^ > σ(*F*^2^) is used only for calculating *R*-factors(gt) *etc*. and is not relevant to the choice of reflections for refinement. *R*-factors based on *F*^2^ are statistically about twice as large as those based on *F*, and *R*- factors based on ALL data will be even larger.

### Fractional atomic coordinates and isotropic or equivalent isotropic displacement parameters (Å^2^)

**Table:**

	*x*	*y*	*z*	*U*_iso_*/*U*_eq_	
C1	0.09639 (15)	0.0309 (3)	1.13097 (16)	0.0513 (6)	
C2	0.01484 (17)	−0.0114 (4)	1.13187 (18)	0.0696 (8)	
H2	−0.0264	0.0653	1.1303	0.084*	
C3	−0.0036 (2)	−0.1670 (5)	1.1352 (2)	0.0857 (10)	
H3	−0.0583	−0.1977	1.1349	0.103*	
C4	0.0570 (2)	−0.2781 (4)	1.1388 (2)	0.0910 (10)	
H4	0.0439	−0.3846	1.1412	0.109*	
C5	0.13830 (19)	−0.2336 (3)	1.13877 (19)	0.0710 (8)	
H5	0.1792	−0.3114	1.1414	0.085*	
C6	0.16037 (15)	−0.0779 (3)	1.13499 (15)	0.0495 (6)	
C7	0.25040 (15)	−0.0379 (3)	1.13676 (16)	0.0527 (6)	
H7A	0.2704	0.0563	1.1752	0.063*	
H7B	0.2895	−0.1237	1.1668	0.063*	
C8	0.25400 (15)	−0.0102 (3)	1.03894 (17)	0.0431 (5)	
C9	0.38632 (13)	−0.0232 (3)	0.93782 (15)	0.0375 (5)	
C10	0.39160 (13)	−0.0260 (2)	0.83950 (14)	0.0357 (5)	
C11	0.32888 (15)	0.0383 (3)	0.76029 (15)	0.0481 (6)	
H11	0.2829	0.0956	0.7674	0.058*	
C12	0.33474 (18)	0.0170 (3)	0.67094 (16)	0.0620 (7)	
H12	0.2928	0.0610	0.6178	0.074*	
C13	0.40156 (19)	−0.0683 (3)	0.65957 (18)	0.0655 (8)	
H13	0.4049	−0.0825	0.5988	0.079*	
C14	0.46365 (18)	−0.1331 (3)	0.73773 (19)	0.0641 (7)	
H14	0.5092	−0.1912	0.7302	0.077*	
C15	0.45842 (15)	−0.1119 (3)	0.82721 (16)	0.0485 (6)	
H15	0.5006	−0.1562	0.8801	0.058*	
C16	0.32922 (14)	0.2572 (2)	0.92940 (13)	0.0374 (5)	
C17	0.25051 (15)	0.3294 (3)	0.91822 (15)	0.0440 (5)	
H17	0.2056	0.2701	0.9264	0.053*	
C18	0.23706 (15)	0.4868 (3)	0.89531 (15)	0.0452 (6)	
C19	0.30418 (17)	0.5693 (3)	0.88050 (16)	0.0521 (6)	
H19	0.2965	0.6755	0.8634	0.063*	
C20	0.38237 (16)	0.4971 (3)	0.89061 (16)	0.0531 (6)	
H20	0.4264	0.5551	0.8797	0.064*	
C21	0.39630 (15)	0.3411 (3)	0.91646 (15)	0.0442 (6)	
H21	0.4497	0.2935	0.9250	0.053*	
C22	0.15279 (17)	0.5646 (3)	0.8891 (2)	0.0673 (7)	
H22A	0.1062	0.5181	0.8377	0.101*	
H22B	0.1560	0.6756	0.8772	0.101*	
H22C	0.1422	0.5498	0.9482	0.101*	
N1	0.33979 (12)	0.0969 (2)	0.95831 (12)	0.0430 (5)	
N2	0.11232 (16)	0.1993 (3)	1.12655 (17)	0.0713 (6)	
O1	0.20433 (11)	−0.0556 (2)	0.96539 (12)	0.0599 (5)	
O2	0.32692 (9)	0.07666 (17)	1.04773 (9)	0.0445 (4)	
O3	0.41541 (10)	−0.12978 (17)	0.99348 (10)	0.0489 (4)	
O4	0.16263 (14)	0.2435 (2)	1.08809 (14)	0.0828 (6)	
O5	0.0756 (2)	0.2889 (3)	1.1631 (2)	0.1459 (12)	

### Atomic displacement parameters (Å^2^)

**Table:**

	*U*^11^	*U*^22^	*U*^33^	*U*^12^	*U*^13^	*U*^23^
C1	0.0461 (15)	0.0671 (17)	0.0454 (14)	−0.0063 (13)	0.0217 (12)	0.0015 (12)
C2	0.0453 (16)	0.102 (2)	0.0651 (18)	−0.0020 (16)	0.0237 (13)	0.0064 (16)
C3	0.053 (2)	0.121 (3)	0.086 (2)	−0.034 (2)	0.0293 (17)	0.001 (2)
C4	0.088 (3)	0.087 (3)	0.106 (3)	−0.032 (2)	0.043 (2)	0.0033 (19)
C5	0.073 (2)	0.0679 (19)	0.084 (2)	−0.0031 (16)	0.0420 (17)	0.0096 (15)
C6	0.0469 (15)	0.0653 (17)	0.0411 (13)	−0.0061 (13)	0.0213 (11)	0.0053 (11)
C7	0.0464 (14)	0.0754 (17)	0.0431 (13)	0.0019 (13)	0.0239 (12)	0.0127 (12)
C8	0.0396 (13)	0.0489 (14)	0.0464 (14)	−0.0004 (11)	0.0218 (12)	0.0058 (11)
C9	0.0327 (12)	0.0396 (12)	0.0414 (12)	−0.0078 (10)	0.0144 (10)	−0.0002 (10)
C10	0.0359 (12)	0.0376 (12)	0.0356 (11)	−0.0061 (10)	0.0149 (10)	0.0008 (10)
C11	0.0468 (14)	0.0554 (15)	0.0425 (13)	0.0050 (11)	0.0158 (12)	0.0007 (11)
C12	0.0725 (18)	0.0729 (18)	0.0371 (14)	0.0067 (15)	0.0140 (13)	0.0031 (12)
C13	0.0731 (19)	0.087 (2)	0.0455 (15)	0.0037 (16)	0.0315 (15)	−0.0020 (14)
C14	0.0576 (17)	0.085 (2)	0.0607 (17)	0.0100 (15)	0.0339 (15)	−0.0015 (15)
C15	0.0443 (14)	0.0586 (15)	0.0444 (13)	0.0007 (12)	0.0175 (11)	0.0007 (11)
C16	0.0426 (13)	0.0394 (12)	0.0330 (11)	−0.0025 (10)	0.0166 (10)	−0.0013 (9)
C17	0.0413 (14)	0.0485 (14)	0.0446 (13)	−0.0065 (11)	0.0177 (11)	−0.0041 (11)
C18	0.0514 (14)	0.0436 (14)	0.0395 (13)	0.0020 (12)	0.0140 (11)	−0.0055 (10)
C19	0.0669 (18)	0.0396 (13)	0.0460 (14)	−0.0043 (13)	0.0141 (13)	−0.0030 (11)
C20	0.0577 (16)	0.0493 (15)	0.0561 (15)	−0.0172 (13)	0.0241 (12)	−0.0019 (12)
C21	0.0407 (13)	0.0516 (14)	0.0432 (13)	−0.0041 (11)	0.0182 (11)	−0.0022 (11)
C22	0.0662 (18)	0.0581 (17)	0.0765 (19)	0.0138 (14)	0.0229 (15)	−0.0032 (14)
N1	0.0497 (11)	0.0489 (12)	0.0410 (10)	0.0054 (9)	0.0291 (9)	0.0080 (9)
N2	0.0727 (16)	0.0707 (17)	0.0808 (17)	0.0045 (14)	0.0397 (14)	−0.0011 (13)
O1	0.0552 (11)	0.0786 (13)	0.0482 (10)	−0.0193 (9)	0.0205 (9)	−0.0073 (9)
O2	0.0425 (9)	0.0603 (10)	0.0370 (8)	−0.0055 (8)	0.0217 (7)	0.0027 (7)
O3	0.0581 (11)	0.0457 (10)	0.0421 (9)	0.0055 (8)	0.0159 (8)	0.0084 (8)
O4	0.0943 (15)	0.0726 (13)	0.0992 (15)	−0.0161 (12)	0.0560 (13)	0.0011 (11)
O5	0.182 (3)	0.0902 (19)	0.224 (3)	0.0186 (18)	0.144 (3)	−0.0170 (19)

### Geometric parameters (Å, °)

**Table:**

C1—C6	1.379 (3)	C12—H12	0.9300
C1—C2	1.385 (4)	C13—C14	1.370 (4)
C1—N2	1.453 (4)	C13—H13	0.9300
C2—C3	1.355 (5)	C14—C15	1.373 (3)
C2—H2	0.9300	C14—H14	0.9300
C3—C4	1.353 (5)	C15—H15	0.9300
C3—H3	0.9300	C16—C21	1.373 (3)
C4—C5	1.380 (4)	C16—C17	1.382 (3)
C4—H4	0.9300	C16—N1	1.415 (3)
C5—C6	1.372 (4)	C17—C18	1.373 (3)
C5—H5	0.9300	C17—H17	0.9300
C6—C7	1.501 (3)	C18—C19	1.380 (3)
C7—C8	1.493 (3)	C18—C22	1.500 (4)
C7—H7A	0.9700	C19—C20	1.378 (3)
C7—H7B	0.9700	C19—H19	0.9300
C8—O1	1.182 (3)	C20—C21	1.371 (3)
C8—O2	1.368 (3)	C20—H20	0.9300
C9—O3	1.206 (3)	C21—H21	0.9300
C9—N1	1.365 (3)	C22—H22A	0.9600
C9—C10	1.493 (3)	C22—H22B	0.9600
C10—C15	1.375 (3)	C22—H22C	0.9600
C10—C11	1.383 (3)	N1—O2	1.425 (2)
C11—C12	1.375 (3)	N2—O5	1.205 (3)
C11—H11	0.9300	N2—O4	1.209 (3)
C12—C13	1.367 (4)		
			
?···?	?		
			
C6—C1—C2	123.0 (3)	C12—C13—H13	120.0
C6—C1—N2	120.8 (2)	C14—C13—H13	120.0
C2—C1—N2	116.2 (2)	C13—C14—C15	119.8 (2)
C3—C2—C1	118.6 (3)	C13—C14—H14	120.1
C3—C2—H2	120.7	C15—C14—H14	120.1
C1—C2—H2	120.7	C10—C15—C14	120.7 (2)
C2—C3—C4	120.5 (3)	C10—C15—H15	119.6
C2—C3—H3	119.8	C14—C15—H15	119.6
C4—C3—H3	119.8	C21—C16—C17	120.6 (2)
C3—C4—C5	120.2 (3)	C21—C16—N1	121.1 (2)
C3—C4—H4	119.9	C17—C16—N1	118.19 (19)
C5—C4—H4	119.9	C18—C17—C16	121.5 (2)
C6—C5—C4	121.9 (3)	C18—C17—H17	119.3
C6—C5—H5	119.1	C16—C17—H17	119.3
C4—C5—H5	119.1	C17—C18—C19	117.5 (2)
C5—C6—C1	115.9 (2)	C17—C18—C22	120.4 (2)
C5—C6—C7	119.1 (2)	C19—C18—C22	122.1 (2)
C1—C6—C7	125.0 (2)	C18—C19—C20	121.1 (2)
C8—C7—C6	112.39 (19)	C18—C19—H19	119.4
C8—C7—H7A	109.1	C20—C19—H19	119.4
C6—C7—H7A	109.1	C21—C20—C19	121.1 (2)
C8—C7—H7B	109.1	C21—C20—H20	119.4
C6—C7—H7B	109.1	C19—C20—H20	119.4
H7A—C7—H7B	107.9	C20—C21—C16	118.2 (2)
O1—C8—O2	124.4 (2)	C20—C21—H21	120.9
O1—C8—C7	127.4 (2)	C16—C21—H21	120.9
O2—C8—C7	108.21 (19)	C18—C22—H22A	109.5
O3—C9—N1	121.6 (2)	C18—C22—H22B	109.5
O3—C9—C10	121.3 (2)	H22A—C22—H22B	109.5
N1—C9—C10	116.88 (19)	C18—C22—H22C	109.5
C15—C10—C11	119.2 (2)	H22A—C22—H22C	109.5
C15—C10—C9	116.82 (19)	H22B—C22—H22C	109.5
C11—C10—C9	123.7 (2)	C9—N1—C16	131.76 (17)
C12—C11—C10	119.7 (2)	C9—N1—O2	112.86 (16)
C12—C11—H11	120.1	C16—N1—O2	110.92 (15)
C10—C11—H11	120.1	O5—N2—O4	122.8 (3)
C13—C12—C11	120.6 (2)	O5—N2—C1	118.2 (2)
C13—C12—H12	119.7	O4—N2—C1	119.1 (2)
C11—C12—H12	119.7	C8—O2—N1	112.06 (16)
C12—C13—C14	119.9 (2)		
			
C6—C1—C2—C3	−1.2 (4)	C21—C16—C17—C18	−0.9 (3)
N2—C1—C2—C3	179.5 (2)	N1—C16—C17—C18	176.48 (19)
C1—C2—C3—C4	0.9 (4)	C16—C17—C18—C19	2.2 (3)
C2—C3—C4—C5	−0.2 (5)	C16—C17—C18—C22	−176.5 (2)
C3—C4—C5—C6	−0.1 (5)	C17—C18—C19—C20	−1.5 (3)
C4—C5—C6—C1	−0.2 (4)	C22—C18—C19—C20	177.2 (2)
C4—C5—C6—C7	179.1 (2)	C18—C19—C20—C21	−0.5 (3)
C2—C1—C6—C5	0.8 (3)	C19—C20—C21—C16	1.8 (3)
N2—C1—C6—C5	−179.9 (2)	C17—C16—C21—C20	−1.1 (3)
C2—C1—C6—C7	−178.3 (2)	N1—C16—C21—C20	−178.47 (19)
N2—C1—C6—C7	1.0 (3)	O3—C9—N1—C16	150.7 (2)
C5—C6—C7—C8	97.3 (3)	C10—C9—N1—C16	−35.5 (3)
C1—C6—C7—C8	−83.5 (3)	O3—C9—N1—O2	−2.9 (3)
C6—C7—C8—O1	−21.8 (4)	C10—C9—N1—O2	170.82 (16)
C6—C7—C8—O2	159.1 (2)	C21—C16—N1—C9	−35.1 (3)
O3—C9—C10—C15	−26.3 (3)	C17—C16—N1—C9	147.5 (2)
N1—C9—C10—C15	159.93 (19)	C21—C16—N1—O2	118.89 (19)
O3—C9—C10—C11	146.8 (2)	C17—C16—N1—O2	−58.5 (2)
N1—C9—C10—C11	−26.9 (3)	C6—C1—N2—O5	−148.5 (3)
C15—C10—C11—C12	−0.7 (3)	C2—C1—N2—O5	30.9 (4)
C9—C10—C11—C12	−173.7 (2)	C6—C1—N2—O4	30.3 (4)
C10—C11—C12—C13	0.6 (4)	C2—C1—N2—O4	−150.3 (2)
C11—C12—C13—C14	−0.2 (4)	O1—C8—O2—N1	−4.2 (3)
C12—C13—C14—C15	0.0 (4)	C7—C8—O2—N1	174.84 (16)
C11—C10—C15—C14	0.5 (3)	C9—N1—O2—C8	−87.7 (2)
C9—C10—C15—C14	174.0 (2)	C16—N1—O2—C8	113.1 (2)
C13—C14—C15—C10	−0.2 (4)		
